# The Face Deepfake Detection Challenge

**DOI:** 10.3390/jimaging8100263

**Published:** 2022-09-28

**Authors:** Luca Guarnera, Oliver Giudice, Francesco Guarnera, Alessandro Ortis, Giovanni Puglisi, Antonino Paratore, Linh M. Q. Bui, Marco Fontani, Davide Alessandro Coccomini, Roberto Caldelli, Fabrizio Falchi, Claudio Gennaro, Nicola Messina, Giuseppe Amato, Gianpaolo Perelli, Sara Concas, Carlo Cuccu, Giulia Orrù, Gian Luca Marcialis, Sebastiano Battiato

**Affiliations:** 1 Department of Mathematics and Computer Science, University of Catania, 95125 Catania, Italy; 2 Applied Research Team, IT Department, Banca d’Italia, 00044 Rome, Italy; 3 Department of Mathematics and Computer Science, University of Cagliari, 09124 Cagliari, Italy; 4 iCTLab s.r.l., 95125 Catania, Italy; 5 Amped Software, 34149 Trieste, Italy; 6 ISTI-CNR, via G. Moruzzi 1, 56124 Pisa, Italy; 7 National Inter-University Consortium for Telecommunications (CNIT), 43124 Parma, Italy; 8 Faculty of Economics, Universitas Mercatorum, 00186 Rome, Italy; 9 Department of Electrical and Electronic Engineering (DIEE), University of Cagliari, 09123 Cagliari, Italy

**Keywords:** deepfake detection, transformer networks, deep learning, deepfake reconstruction, deepfake challenge, discrete cosine transform

## Abstract

Multimedia data manipulation and forgery has never been easier than today, thanks to the power of Artificial Intelligence (AI). AI-generated fake content, commonly called Deepfakes, have been raising new issues and concerns, but also new challenges for the research community. The Deepfake detection task has become widely addressed, but unfortunately, approaches in the literature suffer from generalization issues. In this paper, the Face Deepfake Detection and Reconstruction Challenge is described. Two different tasks were proposed to the participants: (i) creating a Deepfake detector capable of working in an “in the wild” scenario; (ii) creating a method capable of reconstructing original images from Deepfakes. Real images from CelebA and FFHQ and Deepfake images created by StarGAN, StarGAN-v2, StyleGAN, StyleGAN2, AttGAN and GDWCT were collected for the competition. The winning teams were chosen with respect to the highest classification accuracy value (Task I) and “minimum average distance to Manhattan” (Task II). Deep Learning algorithms, particularly those based on the *EfficientNet* architecture, achieved the best results in Task I. No winners were proclaimed for Task II. A detailed discussion of teams’ proposed methods with corresponding ranking is presented in this paper.

## 1. Introduction

The term “Deepfake”, refers to images, videos and audio manipulated or created from scratch by machine learning generative models. Common deep learning approaches exploit Generative Adversarial Networks (GAN) [1] to manipulate multimedia data and generate high-quality fake content. This particular forgery technique have been widely employed for malicious purposes such as for pornography and individual humiliation through social networks. Therefore, the need to counteract the illicit use of this powerful technology was born. Recently, the first forensic ballistics approach on Deepfake images has been proposed in [2] where the objective was the reconstruction of media content history by establishing the number of manipulations performed (made through the use of generative models). Several works on Deepfake detection are present in the literature, but unfortunately most of them lack robustness and generalization: they do not work in real-case scenarios. For example, a Deepfake image shared through common platforms such as WhatsApp or Facebook, will be JPEG re-compressed [3], thus reducing the efficacy of detection methods. Therefore, it is necessary to create much more sophisticated and robust algorithms able to solve several Deepfake-related tasks in the wild. To this aim, in this paper, we present the *Deepfake Detection and Reconstruction Challenge* organized at the 21st International Conference on Image Analysis and Processing (ICIAP) Conference (https://iplab.dmi.unict.it/Deepfakechallenge/, accessed on 1 May 2022). The purposes of this challenge was to create (Task I) “in the wild” robust Deepfake detection algorithms and (Task II) methods able to reconstruct the original image from a Deepfake. The entire challenge was focused only on Deepfake images of human faces, given the importance and the dangerousness of this kind of manipulation.

As far as Task I is concerned, it presented a dataset more challenging than usual detection ones, having Deepfake images generated by different state-of-the-art architectures.

Moreover, randomly, a set of samples were manipulated considering one or more “benevolent” attacks such as JPEG compression; scaling; JPEG compression + Rotations + Gaussian blurring, etc.

Detectors achieving high accuracy in this context should demonstrate that they work in real-case scenarios.

On the other hand, Task II had the objective of reconstructing original images from corresponding Deepfakes. Unfortunately, maybe due to the fact that this task has never been addressed by the scientific community, no participant was able to face this task and to propose a solution given the restrictive deadline given (less than one month).

The main contributions of this work are given below:

- A detailed description of the *Deepfake Detection and Reconstruction Challenge*, organized at the 21st International Conference on Image Analysis and Processing (ICIAP);
- The best challenge solutions created by participating teams were included in the paper;
- A new dataset for the deepfake detection task, which turns out to be different from common datasets available in the literature due to its diversity in terms of image size, types of attacks applied, and much more, was proposed;
- Finally, the first dataset covering a task never addressed by researchers in the domain (creating an algorithm able to reconstruct the original image from deepfake) was proposed.

The remainder of this paper is organized as follows: Section 2 presents a brief overview of state-of-the-art Deepfake creation and detection approaches. A detailed description of the challenge is presented in Section 3. Section 4 describes the most interesting participants’ solutions with corresponding ranking and discussion reported in Section 5. Finally, Section 6 concludes the paper with a comments for possible future works.

## 2. Deepfake Literature Overview

The task of detecting images with human faces created by Generative Adversarial Networks (GAN) has attracted significant attention from the research community, as witnessed by some recent surveys on the topic [4,5,6].

The most famous Deepfake creation techniques from the literature are listed in Section 2.1, some of which were employed to construct the datasets for the Challenge described in this paper, while Section 2.2 describes some of the most effective state-of-the-art Deepfake detection algorithms.

### 2.1. Deepfake Creation Methods

The most effective state-of-the-art techniques for creating Deepfakes are based on GANs. Generator (*G*) and Discriminator (*D*) are the two main components of a GAN, which can be trained simultaneously but with different objectives: *G* has to capture the distribution of the training data while *D* has to estimate the probability that a sample comes from the training data rather than *G*. When *G* has achieved its goal, the training procedure will end. In the latter scenario, *D* will no longer be able to distinguish the images generated by *G* from the training data.

**Figure 1:**
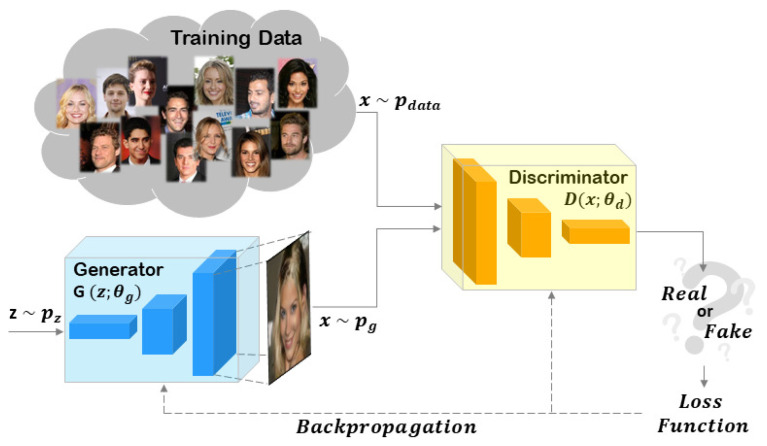
Generic scheme of a GAN architecture. The *Generator* and the *Discriminator* are the main components of the GAN. The objective of the Generator is to capture the data distribution of the training set. The goal of the Discriminator is to distinguish the images coming from the Generator compared to the training data. When the Generator creates images with the same data distribution of the training set, the Discriminator will no longer be able to solve its task and the training phase can be considered completed.

Figure 1 shows a generic scheme of a GAN architecture.

Recently, different GAN-based solutions have been proposed with focus on human faces: several manipulation categories have been introduced: *Entire Face Synthesis* (a person’s face created from scratch) and *Attribute Manipulation* (e.g., change hair color, add glasses, etc.) as an example.

Among all, one of the first effective techniques was the AttGAN, proposed by He et al. [7] with an attribute classification constraint applied in the latent representation of the generated image in order to guarantee only the correct modifications of the desired attributes. Another interesting method is the Group-Wise Deep Whitening and Coloring method (GDWCT), proposed by Cho et al. [8], which is a style transfer approach improving not only computational efficiency but also the quality of the generated images.

Choi et al. proposed StarGAN [9], a framework capable of performing image-to-image translations across multiple domains using a single model. Given a random label as input (such as hair color, facial expression, etc.), StarGAN is able to perform an image-to-image translation task with an impressive visual result. The main limitation of this architecture is that it does not capture the multi-modal nature of data distribution: given an image and label as input, the generator will produce the same output for each domain. This limitation has been overcome by the new StarGAN-v2 architecture [10].

Today, the best entire face synthesis methods, for still images, are indeed the StyleGAN [11] and the StyleGAN2 [12]. The StyleGAN [11] is able to control the style output by mapping points in latent space to an intermediate latent space. The framework is capable of generating impressive photo-realistic and high-quality photos of faces. The main limitations are defined in terms of the overall style of the generated image at different levels of detail. In other words, several artifacts are visible to human eyes on the final generated Deepfake images. These imperfections were fixed through a new version of the generator proposed in the StyleGAN2 [12], resulting in much more realistic faces almost free of anomalies.

### 2.2. Deepfake Detection Methods

Deepfake detection algorithms could be divided into three categories: (i) deep learning-based methods, (ii) physical-based methods and (iii) physiological-based methods. The work proposed by *DC-GAN* (Amped Team) group (who achieved high performance in Task I) belongs to the first category, in that it makes use of a neural network trained to detect GAN images. After the seminal work by Do et al. [13], which was entirely data-driven, other approaches followed where images are pre-processed (e.g., by high-pass filtering [14], or working on the chrominance components [15]) in order to let the network work on a facilitating domain. Several approaches were then proposed [4], and it seemed that, as suggested by Wang et al., most GAN-generated images shared common flaws that made them easy to detect [16]. However, it must be that such flaws were progressively reduced, given that a recent study by Gagnaniello et al. [17] shows that GAN-detection methods are apparently still far from showing reliable performance, especially when tested images that differ significantly from those in the training set.

Audio–visual content generated with Deepfake creation techniques is in some cases virtually impossible to distinguish with the naked eye, and there is now more of a need than ever to develop systems capable of identifying it. However, as shown in [18], the methods for carrying out Deepfake detection are not yet sufficiently accurate and mature, and research will still be needed to achieve satisfactory results. In an attempt to address the problem of Deepfakes detection in videos, numerous datasets have been produced over the years. These datasets are grouped into three generations: the first generation consists of DF-TIMIT [19], UADFC [20] and FaceForensics++ [21]; the second generation datasets includes as Google Deepfake Detection Dataset [22] and Celeb-DF [23]; and finally the third generation datasets include the DFDC dataset [24] and DeepForensics [25]. The further the generations go, the larger these datasets are, and the more frames they contain. Recently, with the increased focus on the concept of identifying Deepfakes ’in the wild’, a number of further important datasets have emerged, namely OpenForensics [26] which seeks to provide images containing multiple faces or crowds of people to address the problem of multi-face forgery detection as also reported in [27] and WildDeepfake [28], which aims to provide a wide variety of scenarios, situations, techniques and perturbations in the images and videos within them. Finally, a particularly large and varied dataset was presented, namely ForgeryNet [29], containing millions of images and hundreds of thousands of videos crafted with dozens of manipulation techniques, perturbations and a great variety of scenes and identities.

In particular, on the DFDC dataset, which is one of the largest and most complete, multiple experiments were carried out trying to obtain an effective method for Deepfake detection. Very good results were obtained with EfficientNet B7 ensemble technique in [30]. Other noteworthy methods include those conducted in [31], who attempted to identify spatio-temporal anomalies by combining an EfficientNet with a Gated Recurrent Unit (GRU). More recently, methods based on Vision Transformers have been proposed. Notably, the method presented in [32] obtained good results by combining Transformers with a convolutional network, used to extract patches from faces detected in videos.

The DFDC dataset was then recently improved by performing distillation from the pre-trained EfficientNet B7 to a Vision Transformer [33]. In this case, the Vision Transformer patches are combined with patches extracted from the EfficientNet B7 pre-trained via global pooling and then passed to the Transformer Encoder. A distillation token is then added to the Transformer network to transfer the knowledge acquired by the EfficientNet B7. The approach proposed by the *AIMH Lab* group to the challenge is a solution based on Vision Transformer.

The latest generation of Deepfakes, especially those generated via GANs, are particularly insidious and affect the reliability of modern multimedia communications [34]. In Deepfakes where imperfections are not visible to the human eye, the image could present non-visible distortions, for example in the frequency domain [35]. In particular, GANs leave a specific “fingerprint”, characterized both by the network architecture (number and type of layers) and its specific parameters [36]. Recently, a number of techniques using the frequency domain have been proposed for the identification of these specific anomalous fingerprints, achieving competitive results [37,38]. Among them are Deepfake detectors based on the concept of Discrete Cosine Transform (DCT): by applying the DCT to the image [39] or utilizing functions extracted from DCT blocks comparable to JPEG compression [40], it is possible to characterize the unique fingerprint of the Deepfake generative architectures. However, realistic scenarios often include some additional transformations applied to the input image. For example, social networks usually resize and compress uploaded pictures to satisfy file size constraints [3]. State-of-the-art models have not been explicitly designed to detect Deepfakes in the presence of additional compression, rescaling and transformations and this can result in a drop in performance if the detectors are used in these conditions. To solve this issue, the *PRA Lab - Div. Biometrics* group proposed a Deepfake detector based on the DCT at different levels of scaling and compression.

In general, Deepfake detection algorithms are focused on detecting anomalies, defined as unique patterns or fingerprints, left by a generative process. In [41,42], the authors proposed solutions capable of capturing a unique pattern left by convolutional layers. Convolutional traces are detected using the Expectation-Maximization [43] algorithm, obtaining features able to distinguish Real from Deepfake images.

Recently, Guarnera et al. [44] demonstrated for the first time in the context of Deepfakes, that it is possible to define the specific GAN model used during the generation process. Specifically, features extracted from the RESNET-18 [45] architecture were compared with a metric learning approach [46]. The authors achieved high performance in the Deepfake Model Recognition task by comparing images generated from 100 different StyleGAN2 models. Additional experiments considering GAN models of different other architectures demonstrated the generalizability of the proposed work, which can be considered as a baseline in this field.

## 3. Deepfake Images Detection and Reconstruction Challenge Description

The aim of challenge participants is to produce new techniques to fight against Deepfake images. For this reason the challenge was divided in two main tasks. In the following sub-sections objectives and evaluation metrics of both have been described.

### 3.1. Deepfake Detection Task

**Figure 2:**
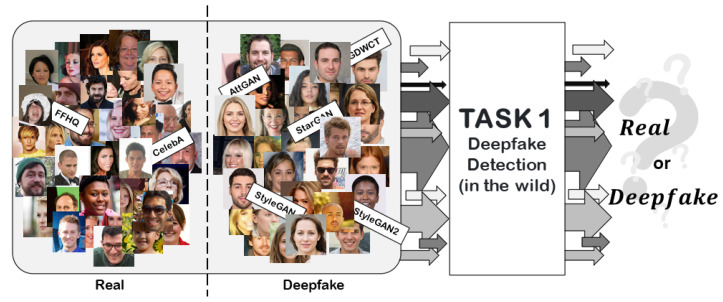
Task I: Deepfake detection task. Given a set of Real and Deepfake images created by different GAN engines, the objective is to create a detector able to correctly classify Deepfake images in any scenario.

In the classic binary classification task for Deepfake detection, participants’ proposed solutions were evaluated with particular emphasis on “robustness” to common image alterations such as: rotation, mirroring, Gaussian filtering, scaling, cropping and re-compressions. Figure 2 summarizes the objective of Task I.

The Deepfake images were generated by several GAN architectures based on well-known Deepfake manipulations such as attribute manipulation and entire face synthesis [6]. The training set was organized into several ZIP files containing images of people’s faces and having the structure “LABEL-GANname.ZIP” (e.g., “0-CELEBA.ZIP”, “1-StarGAN.ZIP”), where LABEL is the Ground Truth (0 if the image is real; 1 if the image is Deepfake). The test set, released in the last part of the competition, was organized as a *TEST*.ZIP file composed by several Real and Deepfake images similar to those of the training set, and in addition, images obtained by applying some processing (rotation, mirroring, etc.) and their random combinations (rotation + mirroring; JPEG compression + scaling, etc.) were introduced.

For this task, the winning team will be selected with respect to the highest classification accuracy value.

Two datasets of real face images were used for the employed experimental phase: CelebA [47] and FFHQ (https://github.com/NVlabs/ffhq-dataset, accessed on 3 November 2021). Different Deepfake images were generated considering StarGAN [9], GDWCT [8], AttGAN [7], StyleGAN [11] and StyleGAN2 [12] architectures. In particular, CelebA images were manipulated using pre-trained models on StarGAN (https://github.com/yunjey/stargan, accessed on 3 November 2021), GDWCT (https://github.com/WonwoongCho/GDWCT, accessed on 3 November 2021) and AttGAN (https://github.com/elvisyjlin/AttGAN-PyTorch, accessed on 3 November 2021). Images of StyleGAN (https://github.com/NVlabs/stylegan, accessed on 3 November 2021) and StyleGAN2 (https://github.com/NVlabs/stylegan2, accessed on 3 November 2021) were created starting from FFHQ dataset. A detailed description of the obtained images is given below:

- CelebA: a large-scale face attributes dataset containing 40 labels related to facial attributes such as hair color, gender and age. The dataset is composed by 178 × 218 JPEG images;
- FFHQ: a high-quality image dataset of human faces. The images were crawled from Flickr and automatically aligned and cropped using dlib (http://dlib.net/, accessed on 3 November 2021). The dataset is composed of high-quality 1024 × 1024 PNG images;
- StarGAN: CelebA images were manipulated by means of a pre-trained template (available in the official repository) obtaining images with a resolution of 256 × 256;
- GDWCT: CelebA images were manipulated by means of a pre-trained template (available in the official repository) obtaining images with a resolution of 216 × 216;
- AttGAN: CelebA images were manipulated by means of a pre-trained template (available in the official repository) obtaining images with a resolution of 256 × 256;
- StyleGAN: images were generated considering FFHQ as the input dataset, obtaining images with 1024 × 1024 resolution;
- StyleGAN2: Images were generated considering FFHQ as the input dataset, obtaining images with 1024 × 1024 resolution.

A preliminary dataset consisting of 500 images from each Real category (CelebA and FFHQ—A total of 1000 people’s faces) and 200 images generated from each Deepfake architecture (a total of 1000 Deepfake images) was collected and shared among participants before the competition began. Once the competition began, a full training set consisting on 5000 images from each Real dataset (for a total of 10,000 real images) and 1000 images from each Deepfake architecture (for a total of 5000 Deepfake images) was released (it did not include images from the preliminary dataset). Both datasets did not contain images with attacks, explicitly listed on the web page dedicated to the competition: rotation, mirroring, Gaussian blur, scaling, cropping and re-compressions.

The test set, released during the last days of the competition, consisted of 7000 images (1000 in each category). Randomly, for each test image, it was determined whether or not to perform the manipulations. In the latter scenario, a random integer number N defined how many manipulations were to be applied to the input data among rotation, mirroring, Gaussian blur, scaling, cropping and re-compressions. In detail, some parameters were set randomly for each involved attack:

- *Rotation*: a random integer number determined the degree of rotation between 45, 90, 135, 180, 225, 270, 315;
- *Scaling*: a random integer number determined whether to reduce the image by 50% or magnify it by 100%;
- *Gaussian Noise*: an integer random number determined the size of the kernel to be applied between [3 × 3, 9 × 9, 15 × 15];
- *Mirror*: a random integer number determined whether to mirror horizontally, vertically or both;
- *JPEG Compression*: a random integer number generated in the range [50, 99] determined the quality factor parameter.

**Figure 3:**
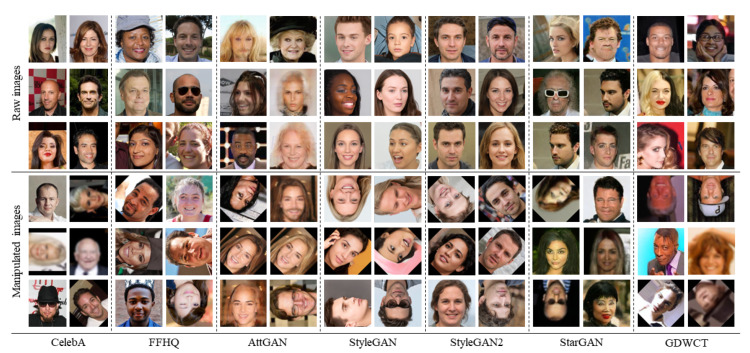
Examples of real (CelebA and FFHQ) and Deepfake images created by different GAN engines (AttGAN, StyleGAN, StyleGAN2, StarGAN, and GDWCT). The columns denote the source of the images. The rows (*Raw images* and *Manipulated images*) show examples of images without and with attacks.

Figure 3 shows examples of raw and manipulated images related to the Task I.

Participants were instructed to send the organizers a text file containing only the estimated labels for each image in the test set. These files were used by the organizers to calculate the classification accuracy and determine the winner of Task I.

### 3.2. Source Image Reconstruction Task

The second task has never been addressed in the literature: given a Deepfake image, the goal was to reconstruct in the best way the source image in its original form starting from the Deepfake counterpart.

**Figure 4:**
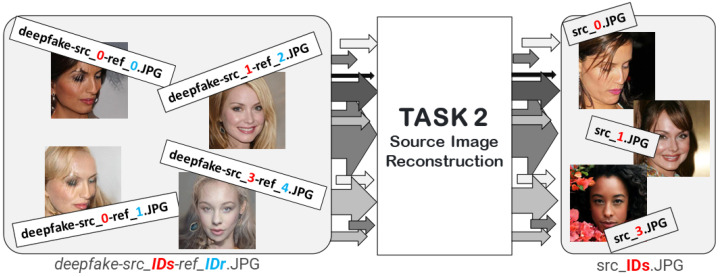
Task II: Source image reconstruction task.

Figure 4 summarizes the objective of the proposed Task.

Each Deepfake sample is obtained through the attribute manipulation operation performed via the StarGAN-v2 [10] architecture on a source image (**src**) with respect to the attributes of a reference image (**ref**).

**Figure 5:**
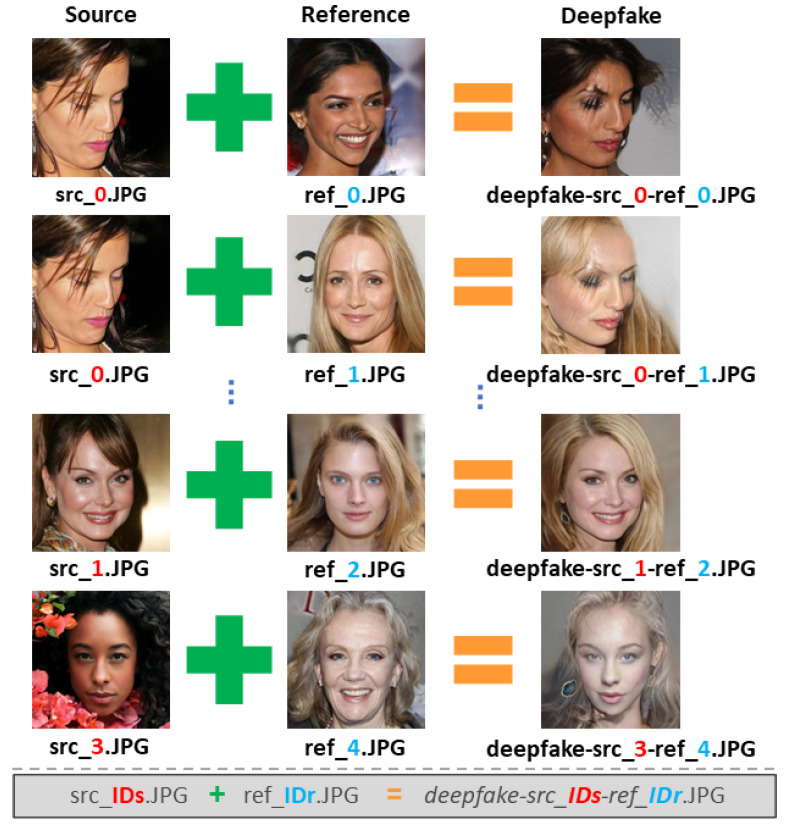
Name structure of source images, reference images and Deepfake images.

The dataset was organized into 3 different ZIP files: *SOURCES*.ZIP, *REFERENCES*.ZIP and *DEEPFAKE*.ZIP. Each Deepfake sample on *Deepfake*.ZIP was named as *Deepfake-src_IDs-ref_IDr*.JPG, where *IDs* refers to the ID of the source image on *SOURCES*.ZIP (with filename *src_IDs*.JPG) and, *IDr* refers to the ID of the reference image on *REFERENCES*.ZIP (with filename *ref_IDr*.JPG). Figure 5 shows some examples of images generated by StarGAN-v2.

For this competition, the winning team was selected with respect to the “minimum average distance to Manhattan” calculated between the sources (available only to the organizers and made public once the competition is over) and the images reconstructed by the participants.

A preliminary dataset consisting of 2000 Deepfake images, 200 source images, and 151 reference images was collected and shared among participants before the competition began. Once the competition began, a full training set consisting of 150,000 Deepfake images, 1500 source images, and 1501 reference images was released. The test set, released during the last days of the competition, consisting of 3000 Deepfake images. No Deepfake image attacks were applied in this context (given the difficulty of the task). Participants were tasked to send the organizers a ZIP file containing only the reconstructed images. These data were used by the organizers to calculate the Manhattan distance and determine the winner of Task II.

## 4. Researcher Solutions

This section shows the best solutions of the participants in Task I. None of the participants created solutions for Task II. A generic discussion of the whole challenge is given in Section 5, where we highlight the main reason why, most likely, none of the participants tried to create a solution for the challenge of reconstructing source images from Deepfakes. Regarding Task I, several teams created solutions based on deep learning, obtaining the best classification results. Interesting and comparative results were also achieved with more analytical approaches.

### 4.1. DC-GAN (Amped Team)

In this sub-section, the DC-GAN (Amped Team) team explains briefly the proposed approach for detecting face images that are generated or synthesized from GANs. The method is deep learning-based, and makes extensive use of data augmentation to improve the robustness and generalization capability of the proposed detector.

As a first step, indeed, participants created an augmented version of the training dataset by applying the following augmentation methods using the Albumentations library [48]:

- *Image compression*: images were compressed with the JPEG algorithm, at a quality factor picked uniformly in the range [50, 99];
- *Noise addition*: images were corrupted with additive Gaussian noise, with variable limit in range [10.0, 50.0];
- *Blurring*: Gaussian blurring was applied to the images, with blur a limit of 3, and sigma limit of 0;
- *Flipping*: both horizontal and vertical flipped versions of each image were generated;
- *Resizing*: images were scaled by the following size [180, 256, 300, 384, 512];
- *Random blackout*: a region around the mouth, nose or eyes was randomly replaced with a black rectangle.

Oversampling with combining augmentation were applied to balance and increase the size of the given dataset. A total of 5 augmented images were created per real input, and 10 augmented images per fake input. Starting from the 10K real images and 5K fake images in the given training set, the dataset was thus increased to 115K images. The dataset was divided into training, validation and testing subsets with ratios 0.7, 0.15 and 0.15, respectively.

**Figure 6:**
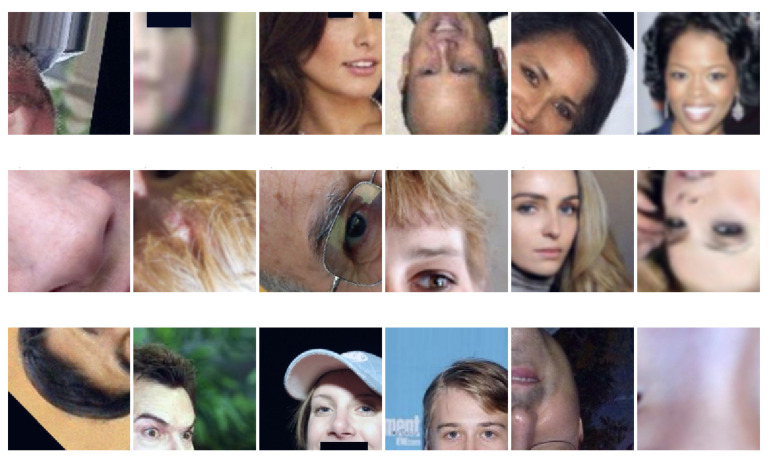
Sample output of random-crop with size 128 × 128.

The team trained and tested the prediction on 64 × 64 or 128 × 128 crops. For training and validation, each image was randomly cropped with a certain size, and normalized to the range [−1, 1] before feeding to the model. During the test phase, the final prediction was made based on the centre-crop image, and four border-crops images. Figure 6 shows some examples of patches obtained with random cropping.

**Figure 7:**
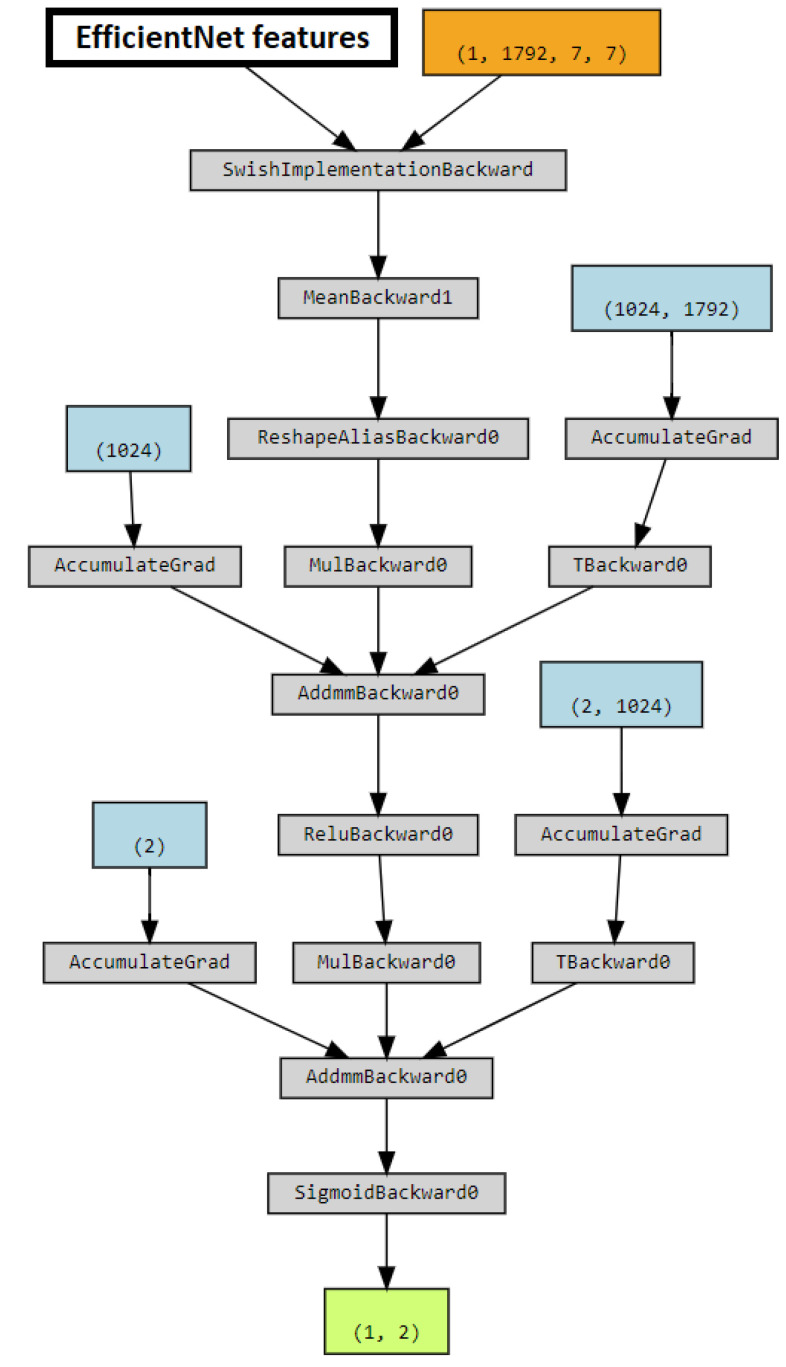
Model employed by the DC-GAN (Amped Team).

The detector was based on EfficientNet [49], with ImageNet pre-trained model. To adapt pre-trained weights to the task, the model was fine-tuned with a small learning rate, then the extracted features were fed to the decision module. In the decision module, the extracted features are obtained by a fully connected layer with output size 1024, and *ReLU* activation. To avoid overfitting, a dropout layer was added before the last fully connected layer. Sigmoid activation is used to return binary classification. The model is shown in Figure 7.

The training was done using Pytorch (pytorch.org, accessed on 1 March 2022) and Pytorch Lightning (pytorchlightning.ai, accessed on 1 March 2022) frameworks with two *RTX 2080 Ti* GPUs. Participants used the Adam optimizer, with an initial learning rate of $10^{-4}$. The learning rate was reduced by a factor of 0.1 if the validation loss did not decrease after five epochs. The last layer’s activation function is Sigmoid, while the loss function is Binary Cross-Entropy, which is defined as:

(1) $$BCE\;loss=-\frac{1}{N}\sum_{i=1}^{N}\left(log\left(p_{i}\right)\right).$$

The testing batch sizes were 32, 64 and 128. Classification results achieved using various version of EfficientNet (B0, B4, and B5) and various sizes of the input image patch (64, 128) were reported. The best results are achieved using model EfficientNet-B5 and for input images of size 128×128 pixels.

**Figure 8:**
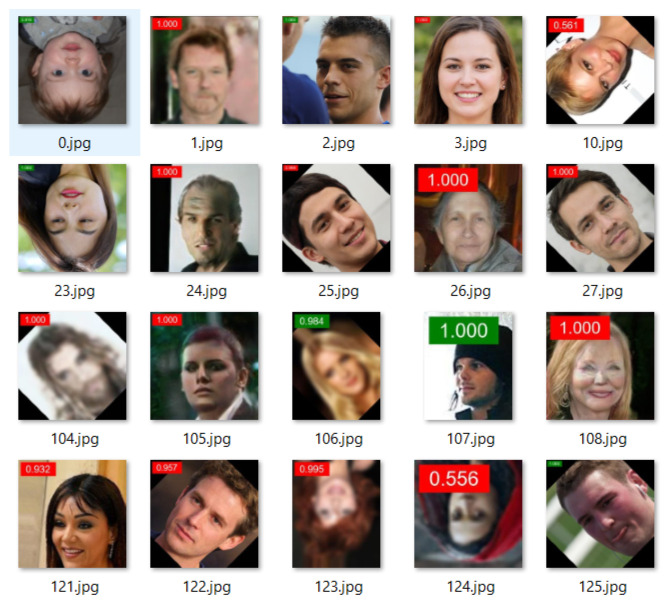
Sample of output results with confidence score: the red label is fake, and green label is real.

Table 1 clearly shows that the best results are achieved using model EfficientNet-B5, and for input images of size 128 × 128 pixels. This is not surprising, considering that EfficientNet-B5 is a richer model, and that larger images contain more data to guide the classification. The chosen model is therefore based on the EfficientNet-B5 model, with input size 128 × 128 and the initial learning rate is $10^{-4}$. The AUC achieved with this configuration on our tests is 0.9674. Figure 8 shows results for some test images obtained with the chosen model.

### 4.2. Convolutional Cross Vision Transformer—AIMH Lab Team

The proposed approach seeks to overcome limitations present in other, more traditional Deepfake detection methods while exploring and effectively exploiting synergies between Convolutional Neural Networks and Vision Transformers [50]. In more detail, the approach presented was designed to address the following objectives:

- To build a leaner architecture than previous approaches in order to apply it more easily on a large scale;
- Simplifying Vision Transformer training by exploiting the inductive biases inherent in Convolutional Neural Networks;
- Construct an architecture capable of analyze an image with a local-global vision;

The proposal analyzes the faces in the dataset to determine whenever they have been manipulated. Having conducted initial evaluation experiments on video datasets, for the DFDC and FaceForensics++ datasets, faces are pre-extracted from the frames using a state-of-the-art face detector, i.e., MTCNN [51]. As the images provided in the challenge dataset are already facial cutouts of the people, this last step was not applied for this specific dataset. Inspired by previous work [27], participants proposed a mixed architecture between a Cross Vision Transformer [52] and an EfficientNets [53,54]. When working with Vision Transformers, the first step is to split the input image into several non-overlapping patches of equal size. This is normally done automatically by simply making a kind of grid and dividing the image into small pieces. In the proposed approach, the AIMH Lab team decided instead to replace this step with a convolutional backbone, which, in addition to being trainable unlike the static traditional approach, performs a transformation of the input image instead of a simple split. Passing through the convolutional layers, in fact, the large three-channel image is transformed into N small images that then represent the input patches to the Vision Transformer. In this case, we chose to use some networks from the EfficientNet category as convolutional backbones. The choice of these specific convolutional backbones stems from the observation that the EfficientNet category seems to have been particularly effective on the Deepfake Detection task in previous works [27,30,33]. Other research work has, however, used larger versions of the EfficientNet, such as the EfficientNet-B7, but since the aim in this case was also to obtain an architecture that was as light as possible, we opted for lighter alternatives for the experiments, namely the EfficientNet-B0 and the EfficientNetV2-M.

**Figure 9:**
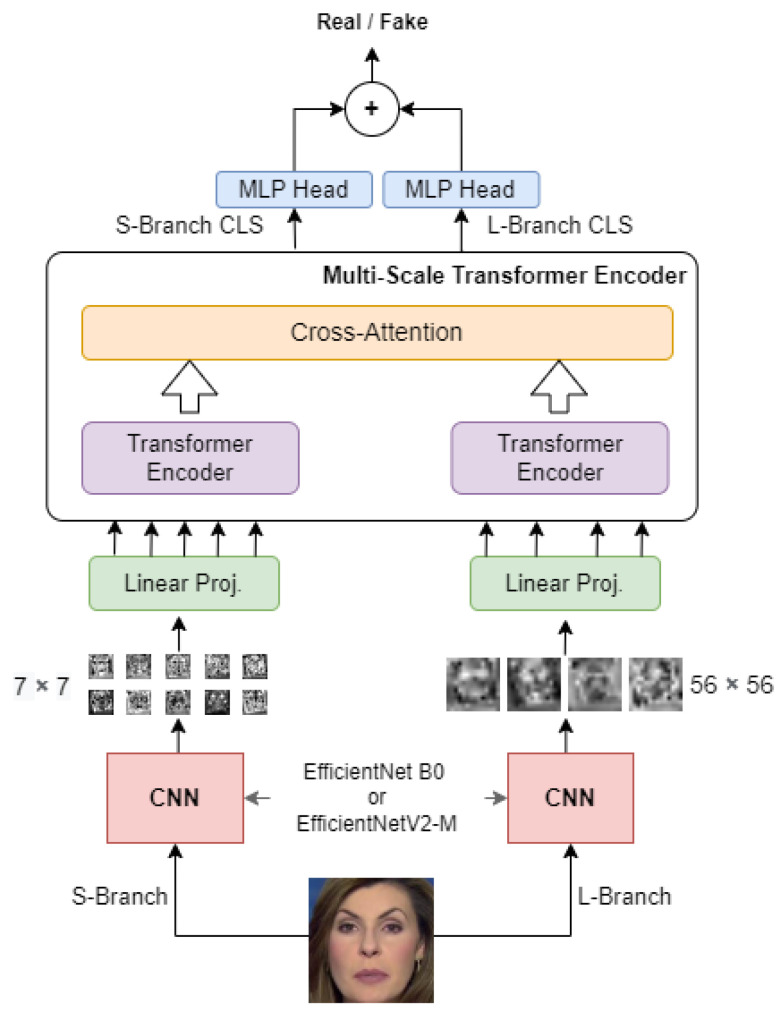
Convolutional Cross ViT architecture.

Using a traditional Vision Transformer, however, means limiting the network to analyze patches of a specific size which may be a huge limitation. For that reason, the proposed architecture is composed of two distinct branches: the *S-branch*, which deals with smaller patches, and the *L-branch*, which works on larger patches to provide a wider receptive field. As shown in Figure 9, the visual tokens output by the Transformer Encoders from the two branches are combined through cross attention, allowing direct interaction between the two paths. Only the two CLS tokens coming out of the transformer encoders are considered for the final classification. These are special tokens added for the purpose of accumulating global information during encoding. The CLS tokens corresponding to the outputs from the two branches are used to produce two separate logits. These logits are summed, and a sigmoid produces the final probabilities. Using the two-way architecture, it is possible to analyse patches of size 7×7 in the S-Branch but also patches of size 56×56 in the L-Branch also exploiting the expressiveness of features extracted from EfficientNets. Two distinct EfficientNet of the same type are used in the two branches as patch extractors.

In conclusion, the resulting architecture is lighter than those based on EfficientNet B7 or ensemble techniques, capable of searching for image anomalies both locally and globally, and also of exploiting the peculiarities of Vision Transformers and EfficientNet in a synergic manner.

### 4.3. Deepfake Detection Using the Discrete Cosine Transform on Multi-Scaled and Multi-Compressed Images—PRA Lab—Div. Biometria Team

This proposed Deepfake detector, presented in [55], is based on a Discrete Cosine Transform (DCT) representation of Deepfake and original images at different scaling and compressing levels. In fact, due to the mismatch between training and testing conditions, state-of-the-art Deepfake models could not provide satisfactory results in a realistic scenario. To work effectively in the detection of artifacts on images that have undergone common transformations due to the use of different software, the team create a tensor of features that is fed into a custom Convolutional Neural Network (CNN), combining information from the original image at different transformation levels. The proposed method consists in splitting a face image in different 8 × 8 pixel blocks with stride 2 × 2, in order to increase the amount of collected information also with small images. Then, for each block the DCT is calculated. The formula used is shown in (2) in the 2D shape (usually known as DCT Type-II) [56]. Starting from each pixel *I*[*x*, *y*] with coordinates *x*, *y* we obtain *F*[*u*,*v*] through:

(2) $$F\left[u,v\right]=\frac{1}{4}C\left(u\right)C\left(v\right)\left[\sum_{x=0}^{7}\sum_{y=0}^{7}I\left[x,y\right]cos\left(a\right)cos\left(b\right)\right]$$

where:

$$\begin{array}{c} a=\frac{(2x+1)u\pi }{16},b=\frac{(2y+1)v\pi }{16},\\ C\left(u\right)=\left\{\begin{array}{cc} \frac{1}{\sqrt{2}} & u=0\\ 1 & u>0 \end{array}\right.,C\left(v\right)=\left\{\begin{array}{cc} \frac{1}{\sqrt{2}} & v=0\\ 1 & v>0 \end{array}\right. \end{array}$$

and

- *u* is the horizontal spatial frequency;
- *v* is the vertical spatial frequency;
- C is a normalising scale factor (orthonormality).

This approach follows the same pipeline as the JPEG compression method with a difference: in this case, we operate an overlap of blocks (stride = 2). Statistics computed from DCT coefficients are then exploited to describe the input data. The DCT coefficient at position (0,0) is called DC and represents the average of the values in the block, whereas the other elements, namely AC, correspond to specific bands of frequencies and can be modelled as a zero-centred Laplace distribution [57]:

(3) $$f\left(x\right)=\frac{1}{2\beta }\exp\left(-\frac{\left|x\right|}{\beta }\right)$$

With β scale parameter computed by MLE (maximum likelihood estimation) close form solution.

The formula used to compute the β is the following:

(4) $$\beta =\frac{1}{N}\sum_{i=1}^{N}\left|x_{i}\right|$$

where *N* is the number of image blocks and $x_{i}$ are the AC values of *i*-th block of the image. Taking all AC coefficients into consideration, 63 β values can be computed for each image. All the features were then collected into a 8 × 8 matrix as follows:

(5) $$B=\left[\begin{array}{cccc} 0 & \beta_{0,1} & \cdots & \beta_{0,7}\\ \beta_{1,0} & \beta_{1,1} & \cdots & \beta_{1,7}\\ \vdots & \vdots & \ddots & \vdots \\ \beta_{7,0} & \beta_{7,1} & \cdots & \beta_{7,7} \end{array}\right]$$

The DC value that does not follow the Laplace Distribution has been set to zero.

The normalization $L_{1}$ and $Z_{Score}$ were carried out on β-values.

**Figure 10:**
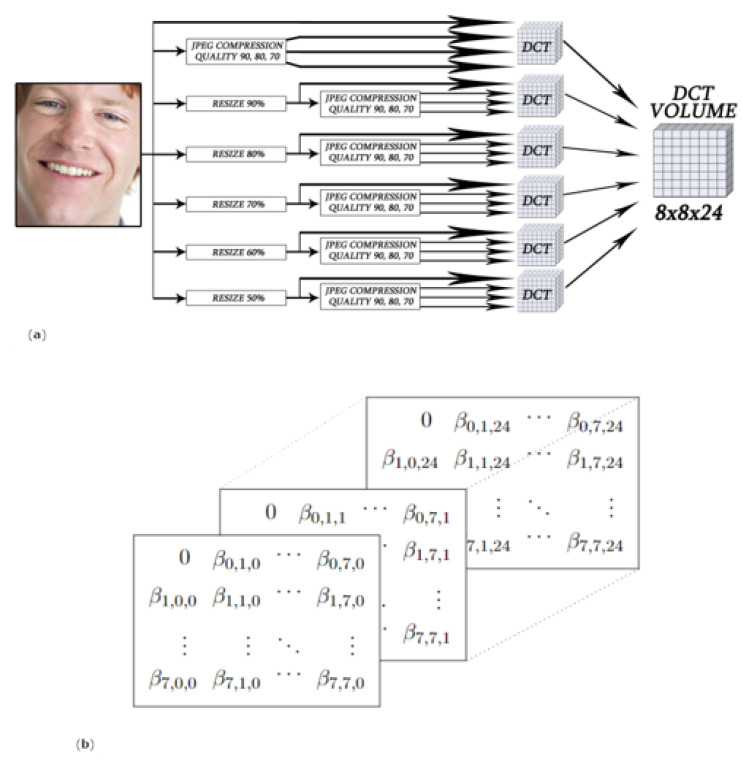
Construction of the representation used as input of the model, designed to be robust to resizing and compressions typical of Deepfakes exchanged on the web. General overview (**a**) and tensor representation (**b**) [55].

The representation defined in (5) has been extended considering six different resizing uniformly distributed from 50% to 100% and four compressions with QF∈{70,80,90,100}. The process and the tensor representation are schematized in Figure 10. The resulting representation was used to train a custom Xception network.

In order to improve the results in presence of resizing and compression, an augmentation process has been also employed.

For each real and fake image, the augmentation was carried out as a single or a combination of the following transformations:

- *Gaussian filters: 3×3, 9×9, 15×15* with σ = 3;
- *Rotation*: 45, 90, 135, 180, 225, 270, 315 degrees;
- *Flip*: vertical flip, horizontal flip or both;
- *Resize*: 50% downscaling, 50% upscaling;
- *JPEG compression*: JPEG compression version at quality 95%.

This tensor representation of Deepfake images that combines the original image’s characteristics at different scaling and compression levels has allowed the team to obtain a high expressive power that can be used to generalize to different application contexts and recognize artifacts over the entire image.

This method performed well on state-of-the-art data: for example, on the OpenForensics [26] dataset, it obtained 99.40% accuracy. However, further manipulations of test data such as compression and resize affect performance. For example, a resize of 55% of the OpenForensics test images leads to an accuracy of 89.30%.

**Figure 11:**
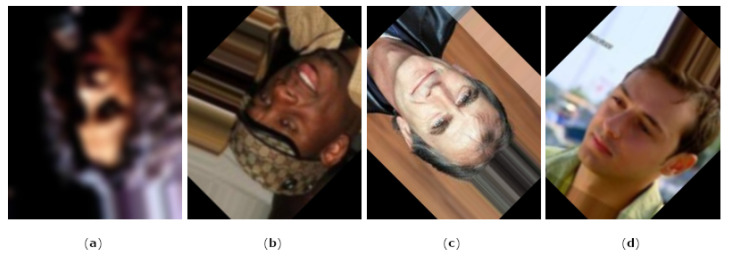
Real samples classified as fake by the “PRA Lab—Div. Biometria” method. In particular: (**a**) 1420.jpg, (**b**) 1794.jpg, (**c**) 3938.jpg, (**d**) 4184.jpg and other real images from the competition dataset contain manipulations external to the facial region that affect the detector.

As for the challenge test, this approach misclassified live samples that contained manipulations outside the face region (Figure 11). In fact, it examines the entire spectrum of the image and detects manipulations even if present in the image’s background or the subject’s hair: the method’s purpose is to detect images that have been manipulated to bully, harass or persecute a victim. The assumption is that such harassment can be perpetrated even with changes localized outside the face region. For this reason, although for the purposes of the competition this portion of samples is considered incorrectly classified, in a real application context this functioning could be useful for detecting manipulations in multimedia files representing an individual.

## 5. Ranking and Discussion

Most researchers participated in the Deepfake Images Detection and Reconstruction Challenge. Only seven teams submitted a solution for Task I and some of them are reported in this paper (only the solutions with classification accuracy values above 60% and only all the teams that actively participated in the ICIAP conference). Table 2 summarizes the classification accuracy scores of the submitted solutions related to Task I. The winning team was VisionLabs, a team composed of Nikita Koritsky and Aleksandr Parkin, who employed an EfficientNet architecture [49] (specifically EfficientNet B3) with pre-trained weights on ImageNet. During training, various preprocessing such as scaling, JPEG compression from 45 to 100 with probability 0.5, hue saturation, gray transformation with probability 0.2, and many others were applied. To increase the robustness of CNN against various types of corruption, this team used rather severe increments, including JPEG compression and Gaussian blurring with large kernels. Pre-trained torchvision models were used in the experiments and tuned for 15 epochs with the Radam optimizer [58]. The learning rate was set to 0.001 and the batch size was set to 32. In the inference step, a threshold obtained on the validation set was used to binarize the obtained values. An Equal Error Rate (resulting in 0.26) was used to calculate the threshold.

Table 3 shows in detail the results obtained by the participants concerning Task I. *Precision*, *Recall* and *F1-score* values for each class are shown. In addition, the *macro average*, defined as the mean of the unweighted mean per label, and the *weighted average*, defined as the mean of the weighted mean of the media per label, are reported. Table 3a–g show all the results of the metrics listed above. These tables are shown sorted with respect to the ranking in Table 2. Let us analyze the results obtained from the solutions proposed by the participants and reported in this paper. In Table 3 we note that all methods are able to define very well whether a multimedia content is a deepfake (just note all the precision, recall and F1-score values). The classifiers implemented by the participants mostly suffer in labeling an image as real. Of course, one must consider that the results shown in the tables are those obtained by testing the classifiers with images in which attacks (such as JPEG compression or combinations of various filters) might be present. These alterations destroy those patterns (such as frequencies or generic traces left by the generative process) present in the multimedia data that were most likely learned by the classifiers. Therefore, if a model has not been trained considering images with classical manipulation attacks, classification performance degrades as shown in the tables. Classification performance was found to be high with the solutions of the first two participants (Table 3a,b). In fact, the authors applied augmentation operations to the training data with attacks similar to those described in the competition. As a result, these classifiers prove to be robust to the most common attacks, such as simple JPEG compression or scaling performed by different social networks (such as Facebook, Instagram).

Regarding Task II, no team submitted a solution. Compared to the creation of a Deepfake Detection algorithm, in this context, there are no baseline works on which participants could study and take into consideration to create a new algorithmic solution or try to improve the results. Consequently, this task had to be analyzed and addressed from scratch, and in addition, the timelines established for submitting a solution during the competition turned out to be very short. The dataset for Task II is available on the web page dedicated to the competition. A supervised learning approach could be a starting point for reconstructing the source image of Deepfakes. Given the nature of the dataset made available for Task II, the Deepfake image and the related source image could be considered the input of a generative model. A basic autoencoder could be used as the basic generative model. A metric capable of calculating the difference between the reconstructed image and the source image (such as the Structural Similarity Index (SSIM) metric) would need to be defined as the loss function. What has just been defined could be considered as a first solution in this area ever addressed by researchers in the field. Having obtained a baseline, further investigations can be conducted in order to create a generic solution that can work with any kind of data and different kinds of semantics (so not only faces of people).

## 6. Conclusions

In this paper, the main solutions to the Deepfake Images Detection and Reconstruction Challenge have been reported. Several new datasets have been made available in order to create increasingly sophisticated Deepfake detection algorithms able to work in any context. The best results in this domain have been obtained through deep learning-based approaches. Surprisingly high-accuracy results were also obtained with an analytical approach based on DCT analysis, despite the fact that the dataset was found to be very complicated through the introduction of various attacks such as scaling, JPEG compression, rotation, and much more. Similar features were already successfully employed in [40]. All the methods reported in this paper can be considered baselines from which forensic researchers can start to create increasing robust and sophisticated solutions. The scenario turns out to be different with the second task characterizing the challenge. Reconstruction of the original image from Deepfakes is a task that is never addressed in the literature and was introduced and described for the first time in this paper. None of the participants provided a solution to this task because it turned out to be very complicated, and the timelines for delivering a solution established during the competition were very constrained. A supervised learning approach, based on the structure of the proposed dataset, could lead to the first solution in this context being obtained. In forensics, having an algorithm (a baseline), capable of “solving” Task II could prove to be extremely important, as it could be used, for example, to justify and prove that a particular cybercrime was not committed by the individual who was subjected to the attack.

In conclusion, in the legal field, it is very important to be able to prove that a piece of media content has been manipulated. The biggest challenge in court is to prove why that image was classified as Deepfake, specifying the elements that were manipulated. The goal is then to obtain an explanation. This property is demonstrated when one is able to identify well those traces left by generative models as demonstrated by the excellent solutions available in the literature and, in particular, those proposed by the participants in this paper.

## Figures and Tables

**Table 1:** Testing AUC values of models trained with different EfficientNet and cropped-image size.

Model and Crop Size	AUC Value
Enet B0 with cropped-image size 64 × 64	0.8115
Enet B0 with cropped-image size 128 × 128	0.8524
Enet B4 with cropped-image size 128 × 128	0.8758
Enet B5 with cropped-image size 128 × 128	0.9674

**Table 2:** Ranking of Task I. The classification accuracy value is reported (in percentage %) for each team.

Ranking	Team Name	Accuracy (%)
#1	VisionLabs	93.61%
#2	DC-GAN (Amped Team)	90.05%
#3	Team Nirma	75.38%
#4	AIMH Lab	72.62%
#5	PRA Lab—Div. Biometria	63.97%
#6	Team Wolfpack	40.61%
#7	SolveKaro	36.85%

**Table 3:** Detailed results of Task I. Precision, recall, F1 score and their mean values are reported for each team.

(**a**) VisionLabs			
	**Precision**	**Recall**	**F1-score**
***Real***	0.89	0.88	0.89
***Deepfake***	0.95	0.96	0.96
***accuracy***			0.94
***macro avg***	0.92	0.92	0.92
***weighted avg***	0.94	0.94	0.94
(**b**) DC-GAN (Amped Team)			
	**Precision**	**Recall**	**F1-score**
***Real***	0.86	0.78	0.82
***Deepfake***	0.92	0.95	0.93
***accuracy***			0.90
***macro avg***	0.89	0.87	0.88
***weighted avg***	0.90	0.90	0.90
(**c**) Team Nirma			
	**Precision**	**Recall**	**F1-score**
***Real***	0.55	0.80	0.65
***Deepfake***	0.90	0.74	0.81
***accuracy***			0.75
***macro avg***	0.72	0.77	0.73
***weighted avg***	0.80	0.75	0.76
(**d**) AIMH Lab			
	**Precision**	**Recall**	**F1-score**
***Real***	0.52	0.49	0.51
***Deepfake***	0.80	0.82	0.81
***accuracy***			0.73
***macro avg***	0.66	0.66	0.66
***weighted avg***	0.72	0.73	0.72
(**e**) PRA Lab—Div. Biometria			
	**Precision**	**Recall**	**F1-score**
***Real***	0.43	0.76	0.55
***Deepfake***	0.86	0.59	0.70
***accuracy***			0.64
***macro avg***	0.64	0.68	0.62
***weighted avg***	0.74	0.64	0.66
(**f**) Team Wolfpack			
	**Precision**	**Recall**	**F1-score**
***Real***	0.05	0.06	0.05
***Deepfake***	0.59	0.55	0.57
***accuracy***			0.41
***macro avg***	0.32	0.30	0.31
***weighted avg***	0.44	0.41	0.42
(**g**) SolveKaro			
	**Precision**	**Recall**	**F1-score**
***Real***	0.17	0.31	0.22
***Deepfake***	0.59	0.39	0.47
***accuracy***			0.37
***macro avg***	0.38	0.35	0.34
***weighted avg***	0.47	0.37	0.40

## Data Availability

The datasets employed for the challenge described in this paper are publicly available here: https://iplab.dmi.unict.it/deepfakechallenge/, accessed on 1 May 2022.
